# Métastase crânienne d’un adénocarcinome rectal: à propos d’un cas avec revue de la littérature

**DOI:** 10.11604/pamj.2017.26.58.9826

**Published:** 2017-02-01

**Authors:** Hamza Samlali, Zineb Bouchbika, Zineb Bennani, Amina Taleb, Nadia Benchekroune, Hassan Jouhadi, Nezha Tawfiq, Souha Sahraoui, Abdellatif Benider

**Affiliations:** 1 Centre Mohamed VI des Traitements de cancer CHU ibn Rochd, Casablanca, Maroc

**Keywords:** Les métastases osseuses, le cancer colorectal, temporale, Bone metastases, colorectal cancer, temporal

## Abstract

Les métastases osseuses sont généralement d'origine pulmonaire, prostatique, rénale, vésicale ou thyroïdienne chez les hommes. Une origine colorectale est rare. Peu de publications ont décrit ce type de métastases, la localisation la plus fréquente est le rachis axial ou le bassin. La localisation crânio-faciale est exceptionnelle. Nous relatons ainsi le cas d'une observation d'un homme suivi pour un cancer du rectum métastatique avec une métastase osseuse temporale. Notre observation se rapportait à l'histoire d'un homme âgé de 38 ans suivi pour un cancer du rectum initialement opéré par résection antérieur classant la tumeur pT3N0M0. 24 mois après, le patient présentait une exophtalmie gauche révélant un processus tumoral temporal. L'évolution et le contexte étaient en faveur d'une métastase. En conclusion, nous rapportons dans cette observation, un cas exceptionnel d'une métastase osseuse crânio-faciale d'un cancer colo-rectal multi-métastatique qui pourra enrichir les rares données rapportées dans la littérature se rapportant aux métastases osseuses de primitif colo-rectal.

## Abstract (en)

The most common primary sites for bone metastases in men are lung, prostate, kidney, thyroid or bladder. Colorectal origin is rare. Few studies have described this type of metastases; the axial skeleton or the pelvis are the most common metastasis locations. Craniofacial location is exceptional. We here report the case of a 38 years old man treated for metastatic rectal cancer metastasized to temporal bone. He initially had undergone surgical procedure for low anterior resection, tumor was classified as pT3N0M0; 24 months after the patient had left exophthalmos revealing a temporal tumoral process. Evolution and context favoured metastasis. In conclusion, this study reporting an exceptional case of craniofacial bone metastasis from multi-metastatic colorectal cancer will enrich the scarce data reported in the literature related to bone metastases from primary colorectal cancer.

## Introduction

Les métastases osseuses sont très fréquentes surtout dans les cancers de la prostate, poumon, rein, sein et thyroïde [1]. Elles restent rares dans les cancers colo-rectaux: elles représentent entre 3,8 et 10,5% des cancers colo-rectaux selon les séries [2]. Elles apparaissent de façon tardive et dans un contexte de métastases multi-viscérales [1, 3, 4]. Seul certaines observations ont rapportées des cas de métastases osseuses cranio-faciales qui restent exceptionnelles. Nous rapportons une observation d’un patient suivi pour une rechute métastatique d’un cancer colo-rectal, 1 an après fin de traitement, révélé par une exophtalmie gauche suite à une métastase crânienne. Nous discuterons aussi au sein de cette observation les aspects épidémio-clinique et évolutif des métastases osseuses des cancers colo-rectaux.

## Patient et observation

**Figure 1:**
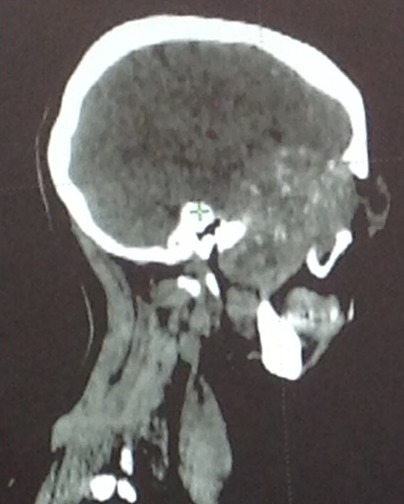
Coupe sagittale d’un scanner cranio-facial montrant un processus tumoral temporal envahissant l’orbite gauche

**Figure 2:**
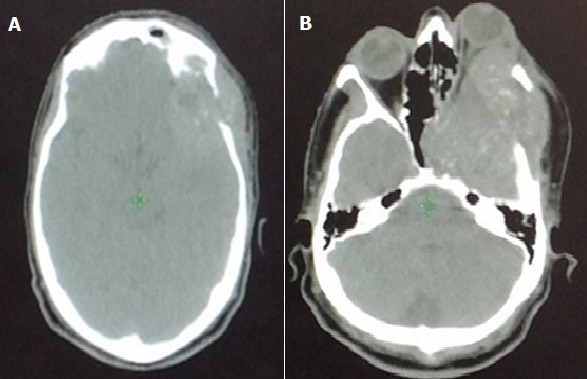
(A et B) coupes transversale d’un scanner cranio-faciale montrant un processus tumoral temporal gauche envahissant l’orbite

Il s’agit d’un patient âgé de 38 ans sans antécédent pathologique particulier qui présentait un syndrome rectal faisant suite à de longs épisodes de constipation. Une examen endoscopique avait révélé une tumeur de la charnière recto-sigmoïdienne. La biopsie était en faveur d’un adénocarcinome liberkhunien moyennement différencié invasif. Le scanner thoraco-abdomino-pelvien (TAP) avait montré un épaississement sigmoïdien sans localisation secondaire hépatique et pulmonaire. Le patient a bénéficié d’une résection antérieur et anastomose colo-rectale. L’analyse macroscopique de la pièce de résection montrait une tumeur ulcéro-infiltrante située à 3.5 de la limite proximale et 13 cm de la limite distale, la tumeur arrivait à la séreuse avec une clearance latérale qui est estimée à 0.6 cm. L’analyse microscopique des 11 ganglions retrouvés ne montrait pas de métastase ganglionnaire. L’analyse microscopique était en faveur d’un adénocarcinome bien différencié classé PT3 N0 M0. Le patient a bénéficié de 6 cures de chimiothérapie adjuvante puis une surveillance régulière par imagerie et dosage des marqueurs tumoraux. 24 mois après la fin du traitement, le patient présentait une exophtalmie gauche avec une baisse de l’acuité visuelle d’apparition progressive associée à une tuméfaction en regard de l’os temporal gauche évoluant vers l’apparition d’un syndrome hypertension intracrânienne sans signe de focalisation. Le scanner cérébrale demandé à la suite de cette symptomatologie, montrait un processus tumoral temporal Figure 1, Figure 2. L’IRM cérébrale montrait un processus sphéno-temporale gauche de 68*52*41 mm assez bien limité sans signe d’extension sur le parenchyme cérébrale avec extension au niveau de la paroi latéral et inférieur de l’orbite. Le scanner TAP montrait une masse pulmonaire à cheval du lobe supérieur et inférieur gauche mesurant 83*82*75 mm associé à une formation surrénalienne gauche de 16*15 mm surrénalienne gauche hypo-dense. Une Ponction-biopsie trans-pariétale de la masse pulmonaire a montré la présence d’un adénocarcinome bien différencié infiltrant et nécrosé dont l’origine secondaire était la plus probable. Le patient a reçu une radiothérapie au niveau de la masse crânienne qui a permis la disparition des céphalées. Le patient a reçu par la suite 3 cures de FolFIri Avastin. L’évaluation montrait une progression de l’exophtalmie gauche avec apparition de localisation hépatique secondaire, progression de la masse surrénalienne et apparition d’une masse musculaire au niveau du mollet droit et le tout dans un contexte de détérioration de l’état général. Vu la mauvais tolérance à la chimiothérapie et l’altération de l’état général (IK<50%), la chimiothérapie a été interrompue et le patient fut inclus dans un programme de soins palliatifs. Le patient est décédé 4 mois plus tard.

## Discussion

Les métastases osseuses des cancers colo-rectaux représentent une entité peu fréquente. La majorité des séries décrivant les métastases osseuses étaient des séries autoptiques [2]. Ces séries rapportaient une fréquence qui variaient entre 1 à 7% [1, 3, 5]. Le cancer rectal est considéré plus ostéophile que le cancer du colon vu qu’on rapporte une fréquence de 3.8 à 10.5% des cas rapportés dans les différentes études, cela comparé à une fréquence de 1 à 3% dans les cancers du colon [1, 2, 5, 6]. L’apparition de ces métastases est généralement tardive dans l’histoire naturelle d’un cancer colorectal connu métastatique [1, 2, 5, 7]. La distribution topographique rapportée dans les différentes publications, montre une atteinte du squelette axiale plus fréquente (rachis et bassin) [8, 9]. La localisation crânienne reste rare et moins fréquente que la localisation mandibulaire. Seul trois cas de localisation crânienne ont été rapportées dans la littérature, Mermillod et al a décris une localisation secondaire d’un primitif colique (1959) [10], trillot et al décris une localisation secondaire frontale d’un primitif rectal (1963) [11] et Delva décris une métastase d’un adénocarcinome colique droit au niveau de l’os temporal (1993) [6]. Ces métastases se développent par voie hématogène, l'invasion osseuse qui se fait par voie hématogène et essentiellement via le plexus veineux para-vertébral de Baston explique la fréquence des atteintes axiales (bassin et rachis lombaire) [1, 12, 13]. La présence d’une connexion entre le plexus ilio-fémoral et les veines lombaires expliquent la fréquence des localisations au niveau du membre inférieur [1, 13, 14]. La présence d’un carrefour orbito-nasal entre la carotide externe et la carotide interne et de nombreuses collatérales de l’artère faciale en regard de la mandibule, de la carotide externe en regard de l’os frontale et de l’artère sylvienne en regard de l’os temporal peuvent expliquer que ces métastases au niveau du massif crânio-facial soit au niveau mandibulaire, temporal, frontale et intra-orbitaire [14]. Ces métastases se manifestent surtout par une symptomatologie non spécifique [13, 15]. Elles se manifestent généralement par: tuméfaction osseuse, douleur, compression nerveuse périphérique, compression médullaire, hypercalcémie [1]. Le cas décris dans cette observation rapportait lui une tuméfaction osseuse avec une exophtalmie. Ces métastases sont exceptionnellement révélatrice d’un cancer colo-rectal [16–18]. Généralement, elle se manifeste de façon métachrone d'un cancer colo-rectal multi-métastatique. Elle apparaît alors avant la cinquième année suivant le diagnostic [19]. Pour la détection de ces métastases en cas de signes d’appel, la scintigraphie osseuse est le meilleur examen [20]. Les aspects radiologiques et tomodensitométriques ne différencient pas les origines colorectales des autres étiologies. Le plus souvent lytiques, elles peuvent revêtir des aspects condensants, voire un aspect pseudo-sarcomateux [20]. L'apparition de lésions osseuses secondaires semble paraître comme un élément péjoratif dans l'histoire d'un cancer colorectal. Des séries font état de médianes de survie de quatre mois [4, 5]. Ce mauvais pronostic conditionne le traitement. Il est donc palliatif, il vise à soulager la douleur et à améliorer la qualité de vie. La radiothérapie est préférée par plusieurs auteurs, du fait de l'impact psychologique d'une amputation, considérée par le patient comme une mutilation [1].

## Conclusion

Les métastases osseuses des cancers colorectaux sont rares. Ces localisations même si peu fréquente sont possible et sont de très mauvais pronostic. Le diagnostic précoce est ainsi important pour améliorer la qualité de vie et la survie du patient.
